# The gapless genome assembly and multi-omics analyses unveil a pivotal regulatory mechanism of oil biosynthesis in the olive tree

**DOI:** 10.1093/hr/uhae168

**Published:** 2024-06-21

**Authors:** Jiaojiao Lv, Chengying Jiang, Wenjun Wu, Kaili Mao, Qianqian Wei, Yuming Zheng, Chengyu Gao, Zhiming Niu, Gaoming Jin, Rong Zhang, Juan Mao, Baihong Chen, Guangpeng Ren, Yongzhi Yang, Dongshi Wan

**Affiliations:** State Key Laboratory of Herbage Improvement and Grassland Agro-Ecosystems, College of Ecology, Lanzhou University, Lanzhou 730000, China; Gansu Research Academy of Forestry Science and Technology, Lanzhou 730020, China; Gansu Research Academy of Forestry Science and Technology, Lanzhou 730020, China; State Key Laboratory of Herbage Improvement and Grassland Agro-Ecosystems, College of Ecology, Lanzhou University, Lanzhou 730000, China; State Key Laboratory of Herbage Improvement and Grassland Agro-Ecosystems, College of Ecology, Lanzhou University, Lanzhou 730000, China; State Key Laboratory of Herbage Improvement and Grassland Agro-Ecosystems, College of Ecology, Lanzhou University, Lanzhou 730000, China; State Key Laboratory of Herbage Improvement and Grassland Agro-Ecosystems, College of Ecology, Lanzhou University, Lanzhou 730000, China; State Key Laboratory of Herbage Improvement and Grassland Agro-Ecosystems, College of Ecology, Lanzhou University, Lanzhou 730000, China; Gansu Research Academy of Forestry Science and Technology, Lanzhou 730020, China; Gansu Research Academy of Forestry Science and Technology, Lanzhou 730020, China; College of Horticulture, Gansu Agricultural University, Lanzhou 730070, China; College of Horticulture, Gansu Agricultural University, Lanzhou 730070, China; State Key Laboratory of Herbage Improvement and Grassland Agro-Ecosystems, College of Ecology, Lanzhou University, Lanzhou 730000, China; State Key Laboratory of Herbage Improvement and Grassland Agro-Ecosystems, College of Ecology, Lanzhou University, Lanzhou 730000, China; State Key Laboratory of Herbage Improvement and Grassland Agro-Ecosystems, College of Ecology, Lanzhou University, Lanzhou 730000, China; College of Horticulture, Gansu Agricultural University, Lanzhou 730070, China

## Abstract

Olive is a valuable oil-bearing tree with fruits containing high levels of fatty acids. Oil production is a multifaceted process involving intricate interactions between fatty acid biosynthesis and other metabolic pathways that are affected by genetics and the developmental stages of the fruit. However, a comprehensive understanding of the underlying regulatory mechanisms is still lacking. Here, we generated a gap-free telomere-to-telomere assembly for *Olea europaea* cv. *‘*Leccino’, representing an olive genome with the highest contiguity and completeness to date. The combination of time-course metabolomics and transcriptomics datasets revealed a negative correlation between fatty acid and flavonoid biosynthesis in the initial phase of olive fruit development, which was subject to an opposing regulatory mechanism mediated by the hub transcription factor MYC2. Multifaceted molecular assays demonstrated that MYC2 is a repressor of fatty acid biosynthesis by downregulating the expression of *BCCP2* (*biotin carboxylase carrier protein 2*), while it acts as an activator of *FLS* (*flavonol synthase*), leading to an increase in flavonoid synthesis. Furthermore, the expression of *MYC2* is regulated by fluctuations of methyl jasmonate content during olive fruit development. Our study completes a high-quality gapless genome of an olive cultivar, and provides new insight into the regulatory mechanisms underlying the biosynthesis of fatty acids and flavonoids in its fruit.

## Introduction

Olive (*Olea europaea* L.), belonging to the Oleaceae family, is extensively cultivated for its oil-rich fruits [1]. Olive oil is renowned for its high concentrations of monounsaturated fatty acids, polyphenols, and other natural bioactive components, which provide a wealth of health-beneficial properties, such as reduced occurrence of cardiovascular disease [2] and cancer [3]. With the increasing consumer demand for olive oil, considerable efforts are constantly being made to breed cultivars that produce high-quality and high-yield oil. However, the selection of olive varieties has historically relied on conventional breeding practices, primarily due to the complexity of oil biochemical pathways [4, 5] and the limited genomic resources available for olives, which makes molecular breeding challenging.

Olive oil is extracted from the fruit, which is botanically classified as a drupe and undergoes a growth period lasting ~5 months from fertilization to ripening [4]. The process of oil biosynthesis consists of fatty acid biosynthesis, glycerol synthesis, and triacylglycerol assembly, in which fatty acids are the building blocks of triglycerides [6, 7]. Recent research has demonstrated that the levels of fatty acids in olive first peaks and then declines in the early stages of fruit maturity, as observed in other plant species [8–10], suggesting that the biosynthesis of fatty acids is closely linked to the process of fruit maturation. During this period, a cascade of biochemical changes occur, involving the biosynthesis and transformation of various metabolites that are closely associated with oil yield, especially fatty acids and flavonoids [4, 9].

Flavonoids have been shown to compete for carbon and energy resources, leading to a reduction in fatty acid biosynthesis in *Arabidopsis thaliana* [11]. Furthermore, flavonoids exert an inhibitory effect on the production of fatty acids by suppressing the expression of *FabG* (*NADPH-dependent 3-ketoacyl reductase*), a key pathway gene in fatty acid chain elongation [9, 12]. Additionally, certain transcription factors that regulate the synthesis of flavonoids, such as Transparent Testas (TT1, TT2 and TT4), also play a role in the inhibition of fatty acid biosynthesis [13–15]. It is evident that reducing the accumulation of flavonoids is a promising avenue for increasing oil yield. However, the regulatory mechanism remains elusive.

High-quality genomes, combined with comprehensive metabolic and transcriptomic maps, present a more exhaustive and systematic view of the interactions and regulatory networks between various metabolites [16, 17]. To date, one wild relative (*O. europaea* var. *sylvestris*), five cultivars (*O. europaea* cv. ‘Farga’, *O. europaea* cv. ‘Arbequina’, *O. europaea* cv. ‘Ayvalik’, *O. europaea* cv. ‘Picual’, and *O. europaea* cv. ‘Leccino’) and one subspecies (*O. europaea* subsp. *cuspidata*) have been sequenced [18–22]. Of these, five varieties have had their genomes assembled using short reads (usually a few hundred base pairs) generated by next-generation sequencing technology, which limits the assembly of long fragments and repetitive sequences. Two genomes (*O. europaea* cv. ‘Arbequina’ and *O. europaea* subsp. *cuspidata*) were assembled through third-generation long-read sequencing, which remarkably improved the quality, but the genomes still exhibit collapsed duplications, gaps, and non-anchored contigs, hindering comprehensive genomic analysis.

Herein, we combined PacBio HiFi (Pacific Biosciences high-fidelity reads), ONT (Oxford Nanopore Technologies) ultra-long reads, and Hi-C (High-throughput Chromosome Conformation Capture) datasets to generate a gap-free telomere-to-telomere (T2T) genome of an essential olive variety (*O. europaea* cv. ‘Leccino’), which is widely cultivated for its high yield, oil quality, disease resistance, and adaptability [23, 24]. The T2T assembly exhibits unprecedented improvements in terms of accuracy, continuity, and completeness compared to the published olive genomes [18–22]. Based on the high-quality genome reference, we analyzed the timing of gene expression and metabolomic profiles during olive fruit development. We detected a negative correlation between fatty acid and flavonoid content in the early stage of olive fruit development. We also revealed that a methyl jasmonate (MeJA)-responsive transcription factor, MYC2, functions as a hub regulator to efficiently balance the biosynthesis of fatty acids and flavonoids. These results have broadened our insights into the regulation of olive oil production.

## Results

### A gap-free telomere-to-telomere genome assembly of *O. europaea* cv. ‘Leccino’

**Figure 1:**
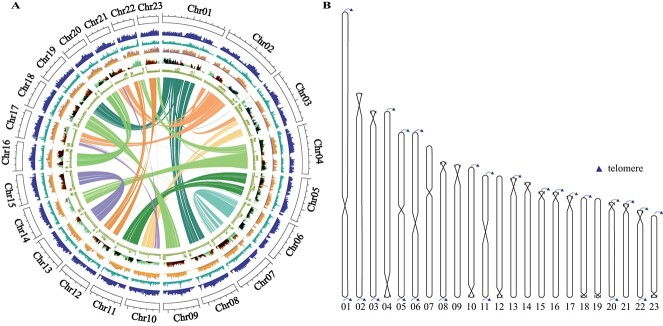
Genomic features of *O. europaea* cv. ‘Leccino’ assembly. **A** Circos diagram of *O. europaea* cv. ‘Leccino’ genome. From inner to outer: intragenome collinear links, LTR coverage, Copia coverage, Gypsy coverage gene density, GC content, and chromosomes. All patterns are illustrated within a window size of 500 kb and chromosome units = 10 000 (kb). **B** Tandem repeat and centromere distribution. Telomeric sequence repeats are indicated by arrows, while the intersection points on the chromosome depict the predicted locations of centromeres.

*Olea europaea* cv. ‘Leccino’ is a widely planted olive cultivar with high-quality oil yield. Here, we applied a synergistic approach, integrating PacBio HiFi reads, ONT ultra-long reads, and Hi-C data, to obtain a high-quality olive genome (Supplementary Data Table S1). The completed assembly encompassed a total size of 1.28 Gb and consisted of 23 chromosomes, exhibiting a scaffold N50 value of 54.85 Mb and a maximum scaffold length of 118.04 Mb (Fig. 1A; Supplementary Data Fig. S1; Supplementary Data Tables S2 and S3). All chromosomes were gapless and all the centromeres were predicted by the quarTeT toolkit [25] (Supplementary Data Table S4). A total of 24 telomeres were identified. For six chromosomes we could identify two complete telomeres (Fig. 1B). In order to further evaluate the quality of the genome, we aligned 72.35 Gb of Illumina short reads to the assembled genome and a high degree of genome coverage (99.67%) was obtained. The high completeness was also evidenced by a 99.93% BUSCO recovery score [26] (Supplementary Data Table S5). The annotation revealed that the total sequences of repeats accounted for 66.30% of the genome, including DNA elements, LINEs, SINEs, and LTRs (Supplementary Data Table S6). Overall, 70 138 protein-encoding genes, 269 microRNAs, 1584 snoRNAs, and 1163 tRNAs were annotated in the genome (Supplementary Data Tables S7 and S8). The final total of 57 737 genes were aligned across various databases including Nonredundant (Nr), TrEMBL, Gene Ontology (GO), and Kyoto Encyclopedia of Genes and Genomes (KEGG), accounting for 82.32% of the total predicted genes (Supplementary Data Table S9).

### Genome evolution and the expansion of fatty acid biosynthesis genes in olive

**Figure 2:**
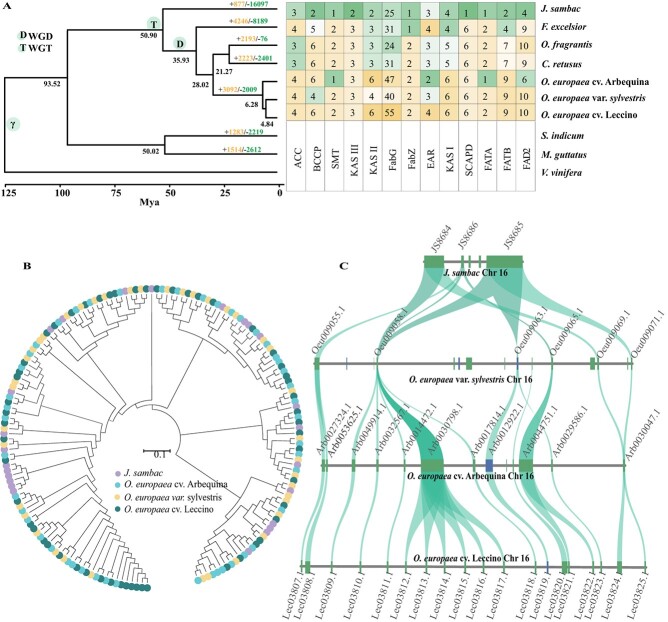
Genome evolution and expansion of fatty acid biosynthesis genes in *O. europaea*. **A** Evolutionary analyses and phylogenetic profiles illustrating the expansions (depicted in light yellow) and contractions (depicted in light green) of orthogroups. These expansions and contractions were determined based on the reconstruction of ancestral gene content at key nodes and the dynamic changes in lineage-specific gene characteristics, and genome-wide identification of fatty acid biosynthesis genes among the Oleaceae family. **B** Phylogenetic tree for FabG proteins in *O. europaea* var. *sylvestris*, *O. europaea* cv. ‘Leccino’, *O. europaea* cv. ‘Arbequina’ and *J. sambac*. The cladistic relationships were determined using the neighbor-joining method with 10 000 bootstrap iterations (see Materials and methods for more information). **C** Collinearity relationship of *FabG* tandem duplication regions between *J. sambac, O. europaea* var. *sylvestris*, *O. europaea* cv. ‘Arbequina’, and *O. europaea* cv. ‘Leccino’. Syntenic blocks are connected by lines. The forward strand of the genome is depicted by the blue bar, while the reverse strand is represented by the green bar.

To examine the phylogenomic relationship of *O. europaea* cv. ‘Leccino’ and other Oleaceae lineages, we selected three varieties of olive (*O. europaea* var. *sylvestris*, *O. europaea* cv. ‘Arbequina’, and *O. europaea* cv. ‘Leccino’), four other species of the Oleaceae family (*Fraxinus excelsior*, *Jasminum sambac*, *Osmanthus fragrans*, and *Chionanthus retusus*), *Sesamum indicum*, *Mimulus guttatus *, and one outgroup (*Vitis vinifera*) to obtain a species tree (Supplementary Data Table S10). A total of 882 single-copy nuclear genes were identified using OrthoFinder (version 2.4.0) software [27]. Then, we obtained a high-support time tree by conducting maximum likelihood and coalescent-based phylogenetic analyses. In the inferred topology, *O. europaea* cv. ‘Leccino’ and its candidate wild species *O. europaea* var. *sylvestris* clustered together, indicating their close relationship. We determined that there are 3092 gene families that have undergone significant expansion in the olive lineage, which were enriched in ‘biosynthesis of secondary metabolites’ processes, particularly those associated with fatty acids (Fig. 2A; Supplementary Data Fig. S2).

We also performed a comprehensive genome-wide analysis of fatty acid biosynthesis genes in the Oleaceae species, and we discovered that most of the pathway genes in olive had largely expanded compared to those in *J. sambac* (Fig. 2A)*.* Using the synonymous substitutions per synonymous site (*K*_s_) distributions of homologous pairs from both intragenomic and intergenomic syntenic blocks as a basis, we revealed that the olive varieties and three other Oleaceae species (*F. excelsior*, *O. fragrans* and *C. retusus*) shared three whole-genome duplication (WGD) events, but only two WGDs occurred in *J. sambac* (Supplementary Data Fig. S3). Most expanded genes of fatty acid biosynthesis gene families in olive originated from the last WGD event, such as *acetyl-CoA carboxylase* (*ACC*), *biotin carboxylase carrier protein* (*BCCP*), *3-ketoacyl reductase* (*FabG*), *hydroxyacyl-ACP dehydrase* (*FabZ*), and *stearoyl-ACP desaturase* (*SCAPD*) (Supplementary Data Fig. S4). Furthermore, subsequent tandem duplication also contributed to the expansion of these genes. Remarkably, most *FabG*s were distributed on chromosome 16 within a 210-kb region constituting the metabolic synthetic gene cluster in the three olive varieties (Fig. 2B and C). The aforementioned findings provided evidence that WGDs and tandem duplications have exerted a substantial influence on the evolutionary trajectory of oil biosynthesis in olive.

### Structural variation identification between different olive varieties

Large-scale genomic alterations spanning over 50 bp, known as structural variations (SVs), can alter gene expression by disrupting the structure or transcriptional element [28–30]. To investigate whether SV affects oil biosynthesis among different olive varieties, we selected four reported high-quality assemblies, namely *O. europaea* cv. ‘Arbequina’, *O. europaea* cv. ‘Farga’, *O. europaea* var. *sylvestris*, and *O. europaea* subsp. *cuspidata* to conduct genomic comparison. Using *O. europaea* cv. ‘Leccino’ as reference, we observed that SVs among individual accessions varied from 27 565 to 43 744. The most SVs were identified between *O. europaea* cv. ‘Arbequina’ and *O. europaea* cv. ‘Leccino’, indicating large genetic divergence between two cultivars (Supplementary Data Fig. S5). A total of 15 082, 9630, 8862, and 8032 functional genes were respectively affected by SVs between *O. europaea* cv. ‘Leccino’ compared with four other olive genomes (*O. europaea* var. *sylvestris*, *O. europaea* subsp. *cuspidata*, *O. europaea* cv. ‘Farga’, and *O. europaea* cv. ‘Arbequina’) (Supplementary Data Fig. 5). Functional enrichment analyses of the SV-influenced genes revealed that they were not related to the terms of fatty acid synthesis and metabolism, but rather associated with hydrolase activity, nucleoside-triphosphatase activity, catalytic activity and cellulose synthase activity (Supplementary Data Fig. S6).

### Integrative analyses of time-course metabolome and transcriptome of fatty acid biosynthesis across the growth of olive fruit

**Figure 3:**
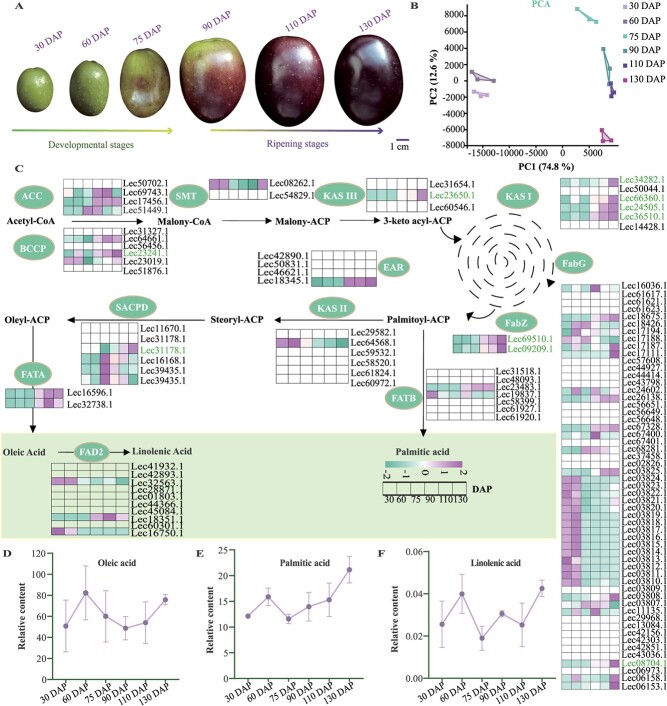
Metabolomic and transcriptomic profiles involved in fatty acid biosynthesis during fruit development and ripening of *O. europaea* cv. ‘Leccino’. **A** Development and ripening of fruits of *O. europaea* cv. ‘Leccino’. DAP, days after pollination. **B** PCA of transcriptome data from six developmental stages of *O. europaea* cv. ‘Leccino’ fruit. ) The KEGG pathway was subjected to an integrated analysis of the transcriptome and metabolome. Each heat map column represents a distinct number of days of fruit development (30–130 DAP). The level of gene expression [log_10_(FPKM +1)] is depicted using a color gradient. ACC, acetyl-CoA carboxylase; BCCP, biotin carboxyl carrier protein; SMT, S-malonyltransferase; KASI, II, and III, ketoacyl-ACP synthase I, II, and III; FabG, ketoacyl-ACP reductase; FabZ, hydroxyacyl-ACP dehydrase; EAR, enoyl-ACP reductase; SACPD, stearoyl-ACP desaturase; FATA, oleoyl-acyl carrier protein thioesterase; FATB, palmitoyl-acyl carrier protein thioesterase; FAD, fatty-acid desaturase. **D**–**F** Oleic acid (**D**), palmitic acid (**E**), and linolenic acid (**F**) concentrations during olive fruit development and ripening.

The production and accumulation of olive oil predominantly occur within the fruit, which undergoes a sequence of physiological, biochemical, and molecular transformations throughout its growth that contribute significantly to fatty acid biosynthesis. To relate the critical stages of fruit development to the key regulators of fatty acid biosynthesis, we performed simultaneous examination of the metabolic and transcriptome characteristics of *O. europaea* cv. ‘Leccino’ throughout the growth of olive fruit (Fig. 3A). Using a UHPLC–MS/MS detection platform, 36 fatty acid-related metabolites were detected, including 30 fatty acyls and 6 lipids (Supplementary Data Figs S7 and S8). Of these, oleic acid was the dominant component, followed by palmitic acid and α-linolenic acid, which together account for ~65% of the total fatty acid content. We found that these three main fatty acids all increased rapidly from 30 to 60 days after pollination (DAP) and then decreased from 60 to 75 DAP. As the fruit ripened, the synthesis of fatty acids resumed, albeit at a slower rate than in the early stages. When the olive was overripe up to the black stage (130 DAP), the accumulation of fatty acids reached a new peak (Fig. 3D–F; Supplementary Data Table S11). This observation aligns with the earlier discovery that the oil content of the fruit first peaks and then declines in the earlier stages of maturity [31].

Additionally, we generated a genetic pathway map consisting of 115 genes implicated in fatty acid biosynthesis, and their corresponding expression levels were determined through RNA sequencing. Forty-seven genes were not detected as transcripts, while 68 genes were differentially expressed at six developmental stages (Fig. 3B and C; Supplementary Data Table S12). We further detected 10 fatty acid biosynthetic pathway genes, including one *BCCP2* (Lec23241.1), one *FabG* (Lec08704.1), two *FabZ*s (Lec09209.1 and Lec69510.1), one *SCAPD* (Lec31178.1), one *KAS III* (*ketoacyl-ACP synthase III*) (Lec23650.1), and four *KAS I*s (*ketoacyl-ACP synthase I*) (Lec34282.1, Lec66360.1, Lec24505.1, and Lec36510.1), which showed a strong association with fatty acid accumulation in the weighted gene co-expression network analysis (WGCNA) [32] (Supplementary Data Figs S9 and S10). Among them, the transcript *BCCP2* (Lec23241.1) was the most highly expressed throughout fruit development, reaching an FPKM of >1000 (Supplementary Data Table S13). These results suggest that this transcript is essential for fatty acid biosynthesis in olive fruit. Similar results were also found in a study in which *BCCP2* expression was reduced by ~38%, leading to an ~9% reduction in fatty acid content in mature seeds of *Arabidopsis* [33]. Subsequently, unraveling the regulatory factors that govern the expression of these pivotal pathway genes holds immense importance in establishing a foundation for improving the quality of oil in olive fruit.

### MYC2 downregulates expression of *BCCP2*

**Figure 4:**
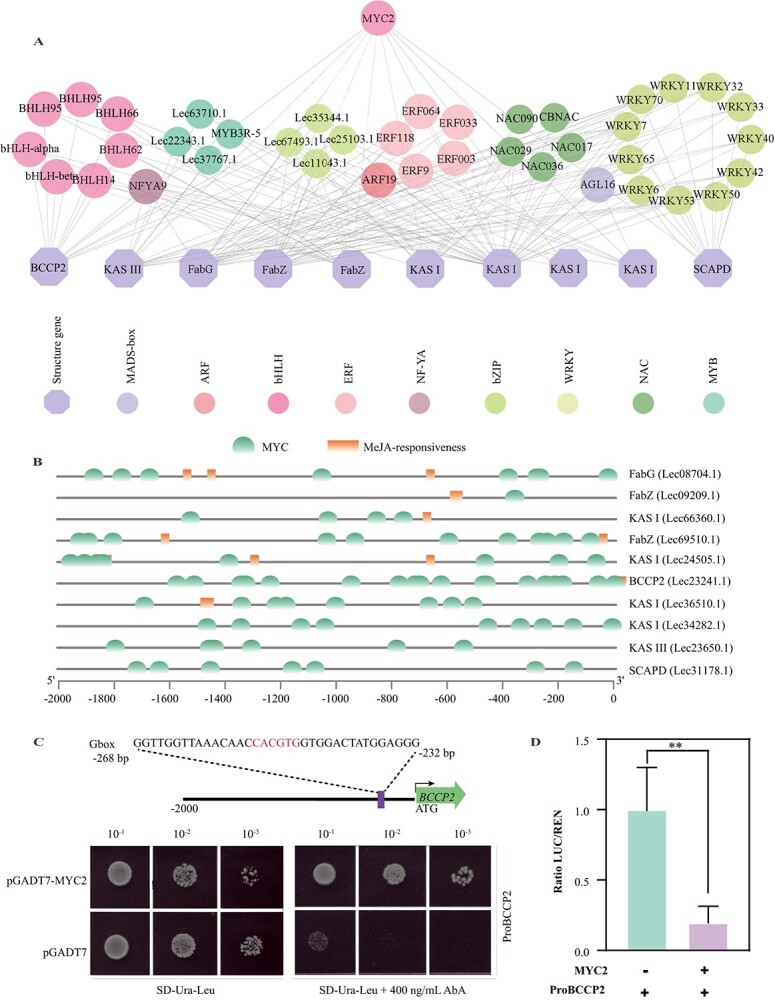
MYC2 directly and negatively regulates the transcription of *BCCP2*. **A** Transcriptional regulatory network of fatty acid biosynthesis genes. Circles of varying colors indicate distinct families of transcription factors (TFs) that have been identified. **B** The analysis focused on identifying MYC binding elements within the promoter regions of 10 genes involved in fatty acid biosynthesis. **C** MYC2 activated *BCCP2*, as indicated by Y1H assays. Yeast cells were genetically modified by introducing a bait vector comprising a fused fragment of the *BCCP2* promoter and an AbAr reporter gene, along with a prey vector containing MYC2 fused to a GAL4 activation domain. To suppress background growth and evaluate the strength of the interaction, a concentration of 400 ng/l aureobasidin A (AbA) was employed. **D** MYC2 reduced the fluorescence intensity of LUC driven by the *BCCP2* promoter compared with the control. The pCXSN empty vector harbored a *ccdB* gene, which acted as a negative selection marker to eliminate the background caused by self-ligation following the transformation process. Results are presented as the mean ± standard deviation of three biological replicates. **P < 0.01 (two-sided Student's *t*-test).

Transcription factors are master regulators of dynamic transcriptional networks that that can interact with specific regulatory elements within the promoter regions of particular genes, enabling them to improve or repress gene expression [34]. Based on gene expression patterns, we identified 84 transcription factors that had a close correlation with the 10 fatty acid biosynthetic genes listed above (Supplementary Data Table S14). Through the integration of *cis*-element analysis, there were 40 transcription factors that may have direct regulatory effects on the 10 fatty acid biosynthesis genes (Fig. 4A). Notably, the promoters of the 10 pathway genes all contained numerous MYC-binding elements (Fig. 4B), suggesting that MYC transcription factors have a pivotal effect on governing the synthesis of fatty acids. Among the candidate transcription factors, *MYC2* (Lec21809.1) was screened, and its expression was negatively related to that of 10 pathway genes.

Given that *BCCP2* (Lec23241.1) exhibited the highest expression levels during olive fruit development (Supplementary Data Table S12), our primary focus was to explore the effect of MYC2 on *BCCP2*. A yeast one-hybrid (Y1H) assay showed that MYC2 could bind to the promoter of *BCCP2* via the element (5′-CACGTG-3′) located between −232 and −268 bp upstream of *BCCP2* (Fig. 4C). To assess the function of MYC2 in the transcriptional activity of *BCCP2*, dual luciferase (LUC) assays were performed. The findings demonstrated a noticeable reduction in the level of LUC expression in *Nicotiana benthamiana* leaves when *MYC2* was co-expressed, compared with the condition without *MYC2* (Fig. 4D; Supplementary Data Fig. S11), indicating that MYC2 has the ability to negatively regulate fatty acid synthesis by directly repressing the promoter activity of *BCCP2*.

### MYC2 upregulates flavonoid biosynthesis by directly activating the promoter of *FLS*

**Figure 5:**
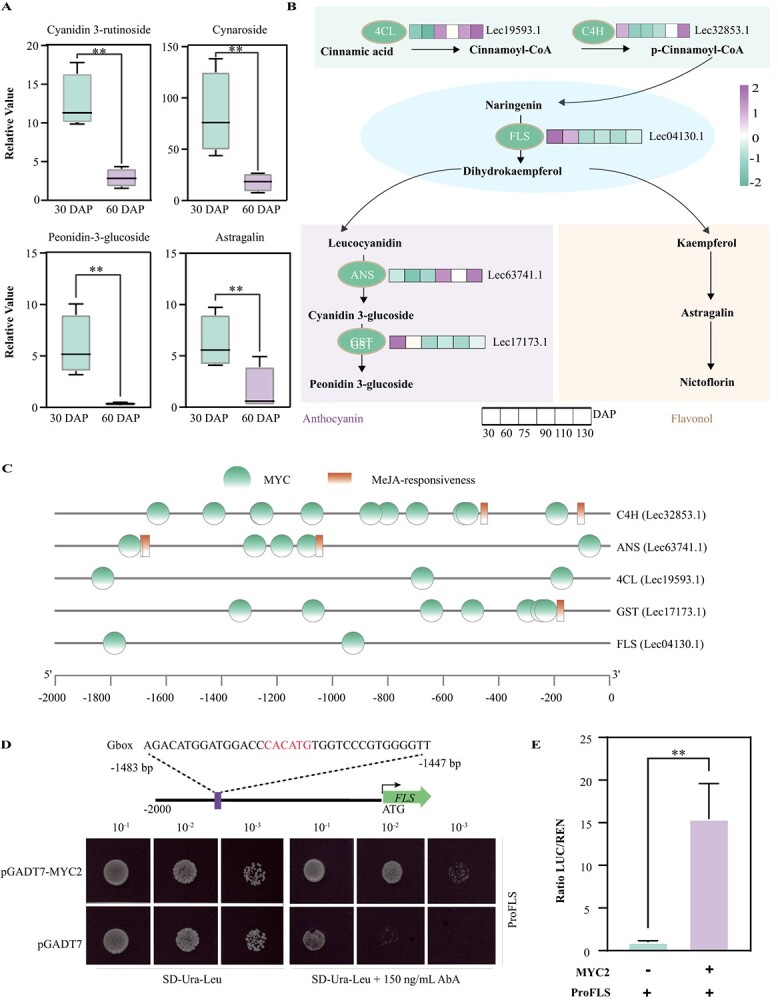
MYC2 directly and positively regulates the transcription of *FLS*. **A** The content of flavonoids undergoes changes during the development and ripening of *O. europaea* cv. ‘Leccino’ fruit. **B** Simplified representation of the flavonoid biosynthetic pathway. The expression value for each gene is indicated in color on a log_10_(FPKM + 1) scale for different numbers of days of fruit development (30–130 DAP). 4CL, 4-coumarate-CoA ligase; C4H, cinnamate 4-hydroxylase; FLS, flavonol synthase; ANS, anthocyanidin synthase; GST, glutathione *S*-transferase. **C** The analysis focused on identifying MYC2 binding elements within the promoter regions of 10 genes involved in flavonoid biosynthesis. **D** MYC2 activated the *FLS* promoter, as indicated by Y1H assays. Yeast cells were genetically modified by introducing a bait vector comprising a fused fragment of the *FLS* promoter and an AbAr reporter gene, along with a prey vector containing *MYC2* fused to a GAL4 activation domain. To suppress background growth and evaluate the strength of the interaction, a concentration of 150 ng/l aureobasidin A (AbA) was employed. **E** Expression of *MYC2* resulted in an increased fluorescence intensity of the LUC reporter gene under the control of the FLS promoter, in comparison with the control condition. The pCXSN empty vector carried a *ccdB* gene, which served as a negative selection marker to eliminate any background caused by self-ligation after the transformation process. Data are presented as the mean ± standard deviation of three biological replicates. ^**^P < 0.01 (two-sided Student's *t*-test).

Apart from being regulated by transcription factors, the biosynthesis of fatty acids is intricately associated with flavonoid biosynthesis. Considerable research has indicated that the reduction of flavonoids could lead to a rise in the accumulation of fatty acids [11, 14, 15, 35, 36]. In line with these studies, we detected a negative correlation between flavonoid and fatty acid synthesis during the early stages of olive fruit development (Supplementary Data Fig. S12). Specifically, a total of 118 flavonoids in olive fruits were identified and mostly consisted of cymaroside (40.66%), followed by rutin (26.63%), cyanidin 3-rutinoside (6.52%), astragalin (3.26%), and peonidin-3-glucoside (1.12%). The contents of major flavonoids substantially decreased from 30 to 60 DAP (Fig. 5A; Supplementary Data Table S11), accompanying the increase in fatty acid synthesis (Fig. 3D–F).

The flavonoid content was basically consistent with the expression of five genes, including *4-coumarate-CoA ligase* (*4CL*), *cinnamate 4-hydroxylase* (*C4H*), *flavonol synthase* (*FLS*), *anthocyanidin synthase* (*ANS*), and *glutathione S-transferase* (*GST*) (Fig. 5B; Supplementary Data Fig. S13). It is noteworthy that the promoters of the aforementioned five genes also contained multiple MYC-binding elements, indicating the possible involvement of *MYCs* in the regulation of flavonoid synthesis (Fig. 5C). Among the five pathway genes, *FLS*, which can catalyze the formation of flavonols from dihydroflavonols, showed the highest transcript accumulation, so we focused on the effect of MYC2 on it. The Y1H assay showed that MYC2 can bind to the *FLS* promoter via the element (5′-CACATG-3′) located between −1447 and −1483 bp in the promoter (Fig. 5D). The dual LUC assay demonstrated that MYC2 can apparently elevate the transcription of the *FLS* promoter, indicating that MYC2 is a positive regulator of flavonoid biosynthesis (Fig. 5E; Supplementary Data Fig. S11).

### Methyl jasmonate regulates the expression of *MYC2* during the growth of olive fruits

**Figure 6:**
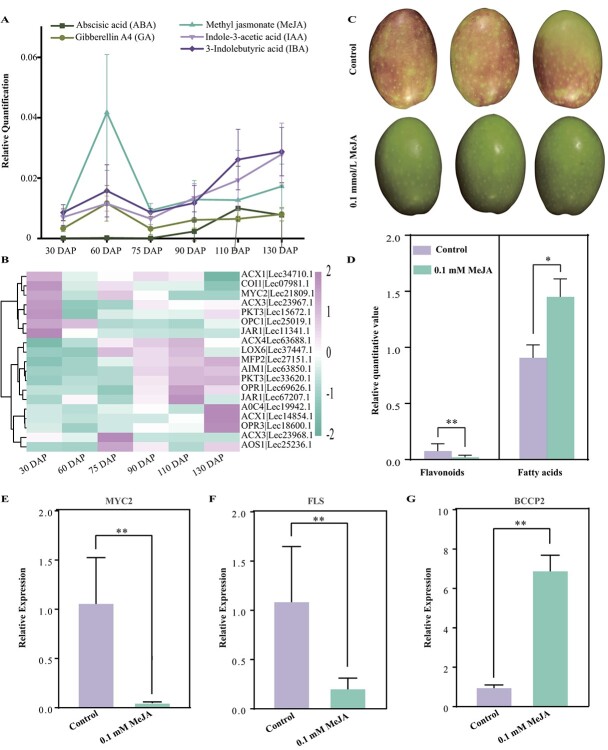
MeJA could inhibit the expression of *MYC2*, thus reducing flavonoid synthesis but increasing fatty acid accumulation. **A** Plant hormones undergo dynamic changes during the development and ripening of *O. europaea* cv. ‘Leccino’ fruit. **B** Expression (FPKM) of major JA-related genes during the development of *O. europaea* cv. ‘Leccino’ fruit. **C** Phenotypic comparison of fruits treated with 0.1 mM MeJA and control fruits (treated with water). **D** Contents of flavonoids and fatty acids in olive fruits treated with or without 0.1 mM MeJA. ^**^P < 0.01; ^*^P < 0.05 (two-sided Student’s *t*-test. **E**–**G** Expression of *MYC2*, *FLS*, and *BCCP2* in control and 0.1 mM MeJA-treated olive fruits.

As demonstrated above, MYC2 functions as a repressor of fatty acid biosynthesis by downregulating the expression of *BCCP2*, while acting as an activator of the production of flavonoids through upregulating the transcription of *FLS* (Figs 4 and 5)*.* We observed that the expression of *MYC2* was markedly suppressed at 60 DAP (Fig. 6B), which is a reasonable explanation for the fact that the accumulation of fatty acids was significantly increased while the production of flavonoids was greatly reduced during this period (Figs 3D–F and 5A). MYC2 is a crucial responder of jasmonic acid (JA) and some other phytohormones, such as auxin (IAA), abscisic acid (ABA), salicylic acid (SA), and gibberellins (GAs) [37, 38]. Our observations revealed that the content of MeJA increased by ~5-fold at 60 DAP relative to 30 DAP, and the levels of IBA (indole-3-butyric acid), GA, and IAA were also elevated, but not significantly. This suggests that the upregulation of MeJA content most likely contributed to the downregulation of expression of *MYC2* (Fig. 6A).

To validate this, we sprayed MeJA (0.1, 1, or 5 mM) uniformly on the surface of immature fruits on the tree. The treated fruits were harvested at the same time as other normal fruits and used for the next analysis. We found that most fruits treated with 0.1 or 1 mM MeJA had less red pigment (anthocyanin) in the pericarp than normal fruits, whereas fruits treated with 5 mM MeJA accumulated more red pigment in the pericarp than the control (Supplementary Data Fig. S14), indicating that varying levels of MeJA may affect flavonoid (anthocyanin) biosynthesis in different ways. Furthermore, we detected that fruits treated with 0.1 mM MeJA displayed a lower level of flavonoids but higher production of fatty acids comparedto untreated fruits, using a targeted global metabolomics platform (GC–MS) (Fig. 6C and D). Consistent with the changes in metabolite levels, the expression of *MYC2* and *FLS* decreased significantly, while the expression of *BCCP2* increased in the 0.1 mM MeJA-treated fruits relative to the control (Fig. 6E–G). These findings suggested that the expression of *MYC2* can be downregulated by the accumulation of MeJA during olive fruit maturation to reduce the biosynthesis of flavonoids while increasing the production of fatty acids.

## Discussion

The yield and composition of olive oil vary with the growing conditions, variety, and maturity of the fruits. A complete reference genome, along with comprehensive transcriptomic and metabolomic profiles, offers tremendous value for genomic research and molecular breeding. To date, seven different olive varieties have been sequenced, generating the genome sizes of 1.31 Gb (*O. europaea* cv. ‘Farga’), 1.1 Gb (*O. europaea* cv. ‘Arbequina’), 0.93 Gb (*O. europaea* cv. ‘Ayvalik’), 0.7 Gb (*O. europaea* cv. ‘Picual’), 0.54 Gb (*O. europaea* cv. ‘Leccino’), 1.14 Gb (*O. europaea* var. *sylvestris*), and 1.19 Gb (*O. europaea* subsp. *cuspidata*), respectively (Supplementary Data Table S2) [18–22]. Although all available olive genomes were assembled to the chromosome level, a large number of non-anchored contigs and gaps ranging from 110 297 to 110 804 712 bp have not been addressed. Using ONT ultra-long, PacBio HiFi, and Hi-C technologies, we constructed a high-quality gap-free assembly of *O. europaea* cv. ‘Leccino’, providing the first opportunity to analyze the telomere and centromere regions (Fig. 1; Supplementary Data Fig. S1). Our assembly exhibits the highest completeness (99.93%) and an unprecedented contig N50 of 54.85 Mb, surpassing all published olive genomes (Supplementary Data Tables S2 and S5). The availability of genomic resources for olive varieties, in particularly the T2T genome assembly, collectedly serves as the solid foundation for further horticulture research.

In plants, the regulation of fatty acid biosynthesis is an intricate network involving multiple metabolic pathways. Prior investigations have indicated that the accumulation of flavonoids could compete for synthesizing substrates and suppress the activity of an essential enzyme, FabG, consequently impeding fatty acid synthesis [9, 12]. In our study, we made the intriguing discovery of a substantial expansion of the *FabG* family in olive that is attributed to WGD events and tandem duplications (Fig. 2; Supplementary Data Figs S3 and S4). This expansion is likely to enhance the activity of FabG reductase, thereby facilitating fatty acid biosynthesis in olive, even in the presence of elevated levels of flavonoids. Furthermore, we constructed holistic transcriptome and metabolome datasets at six different development stages of olive fruit and observed a negative correlation between the synthesis of fatty acids and flavonoids, particularly at the initial stage of maturation (at 60 DAP) (Supplementary Data Fig. S12). During this stage, the production of flavonoids was reduced, while the accumulation of fatty acids was significantly increased (Figs 3D–F and5A).

Co-expression network analysis demonstrated that *MYC2* expression was negatively associated with the accumulation of fatty acids but positively correlated with the flavonoid content (Supplementary Data Tables S11 and S12). MYC2 is a special class of transcription factor that can act as both a transcriptional activator and a transcriptional repressor, differentially modulating diverse jasmonate-dependent functions in plants [37–39]. For instance, MYC2 negatively regulates tryptophan metabolism and glucosinolate biosynthesis, while it contributes to auxin biosynthesis and enhanced resistance to insect herbivory [39]. Recent studies have revealed that MYC2 can form a heterodimer with bHLH60 to induced the biosynthesis of flavonoids in *Salvia miltiorrhiza* [40], and it can directly downregulate the expression of *FAD2* (*Fatty Acid Desaturase 2*) to reduced oleic acid degradation in *Camellia oleifer* [41]. The above findings indicated that MYC2 may function as hub regulator to balance the biosynthesis of fatty acids and flavonoids in plants.

**Figure 7:**
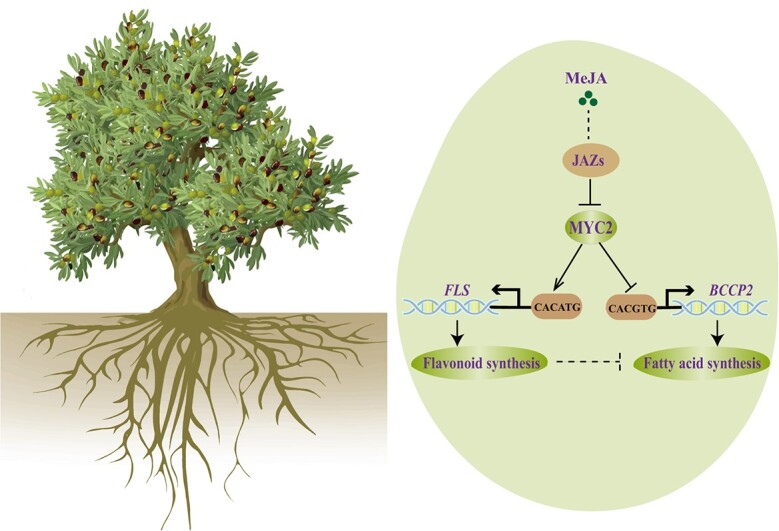
Model of MYC2 in the modulation of fatty acid and flavonoid biosynthesis under MeJA treatment. In the early stage of olive fruit development, accumulation of MeJA could decrease the expression of *MYC2*, which could repress fatty acid biosynthesis by downregulating the expression of *BCCP2* though binding to the G-box within the promoter. Conversely, MYC2 was proved to directly bind to and activate the promoter of *FLS*, ultimately leading to an increase in flavonoid content.

In our study, a negative correlation was observed between the expressions of *MYC2* and 10 fatty acid biosynthesis pathway genes, including one *BCCP2*, *FabG*, *SCAPD*, *KAS III*, two *FabZ*s, and four *KAS I*s (Fig. 4A). On the other hand, we discovered a positive relationship between the expression of *MYC2* and five flavonoid biosynthesis pathway genes, namely *4CL*, *C4H*, *FLS*, *ANS*, and *GST*, in the early stages of olive fruit development (Fig. 5B). MYC2 can bind to the G-box elements, which are DNA sequences with the consensus motif 5′-CACNTG-3′. Specifically, MYC2 exhibits a strong affinity for *cis*-sequences containing 5′-CACGTG-3′ or 5′-CACATG-3′ [37–39]. In this study, we detected that MYC2 has the ability to downregulate the expression of *BCCP2* by binding to the *cis*-element (5′-CACGTG-3′) located between −232 and −268 bp in the promoter region (Fig. 4C and D). Conversely, MYC2 is able to induce the expression of *FLS* by combining with the *cis*-sequence (5′-CACATG-3′) located between −1447 and − 1483 bp in the promoter (Fig. 5D and E). These results confirmed that MYC2 is a hub regulator in the coordination of fatty acid and flavonoid biosynthesis in olive (Fig. 7).

A genome-wide transcript analysis in *Arabidopsis* revealed that MYC2 regulates the expression of target genes predominantly in a JA-dependent manner [42, 43]. We detected that the content of MeJA was highly elevated at 60 DAP in olive fruit, leading to a decline of *MYC2* expression, as observed in the case of pear calli [44] (Fig. 6A and B). Simultaneously, the synthesis of flavonoids was remarkably inhibited, while a notable increase in the content of fatty acids was observed (Figs 1D–F and 5A). Low concentrations of MeJA (0.1 and 1 mM) exogenously sprayed on the early stages of olive fruit development decreased flavonoid (anthocyanin) accumulation, while a higher concentration of MeJA (5 mM) could promote flavonoid production (Fig. 6C and D; Supplementary Data Fig. S14). Similarly, seeds of *Camellia oleifera* treated with 0.1, 0.5 and 2.5 mM MeJA produced higher fatty acid contents compared with the control, while comparatively lower fatty acid contents were detected when the seeds were treated with 5 mM MeJA [41]. These results indicated that the regulation of metabolite synthesis by MeJA is closely related to the treatment concentrations [43, 45–47], which aligns with variation in MeJA content throughout the development of olive fruits.

## Materials and methods

### Genome sequencing, assembly, and annotation of *O. europaea* cv. ‘Leccino’

The roots, fruits, leaves, and xylems of *O. europaea* cv. ‘Leccino’ were sampled from the National Germplasm Repository of Olive of China (Longnan, Gansu, China). The genomic DNA of young leaves was sequenced on the Oxford Nanopore Technologies (ONT) and PacBio SMRT (Single Molecule Real-Time) platforms. The DNA library of short reads was sequenced on the Illumina HiSeq 4000 platform. The PacBio HiFi and ONT ultra-long reads were assembled using Hifiasm (version 0.19.4) software [48]. Then, Purge_haplotigs [49] was utilized for haplotype removal with parameters -l 3 -m 22 -h 85. The Hi-C paired-end reads obtained from the Illumina NovaSeq 6000 platform were applied to dissect the chromosome conformation and spatial location of genomes using Juicer (version 2.041) [50] and the 3d-DNA pipeline (180419) [51]. Finally, Juicebox (version 1.11.08) [52] was used to adjust some position and orientation errors manually. The completeness of assembly was evaluated by Compleasm software [26] using the embryophyta_odb10 database. The repeated sequences and the structure and function of genes were annotated as previously described [29]. The TeloExplorer and CentroMiner modules of the user-friendly toolkit quarTeT [25] were used to identify candidate telomeres and centromere regions.

### Phylogenomic and whole-genome duplication analyses

We used the OrthoFinder (version 2.4.0) [27], MAFFT (version 7.471) [53], PAL2NAL (version 14) [54], IQ-TREE (version 2.1.2) [55], and ASTRAL (version 5.7.5) [56] pipelines to construct a phylogenomic tree of three varieties of olive (*O. europaea* var. *sylvestris*, *O. europaea* cv. ‘Arbequina’, and *O. europaea* cv. ‘Leccino’), four other species of the Oleaceae family (*F. excelsior*, *J. sambac*, *O. fragrans*, and *C. retusus*), *S. indicum*, *M. guttatus* and *V. vinifera*. The outcome maximum likelihood tree and four fossil calibration times (*J. sambac*–*V. vinifera*, 112.4–125.0 Mya; *O. fragrans*–*S. indicum*, 56.2–74.2 Mya; *J. sambac*–*F. excelsior*, 38.1–51.2 Mya; *F. excelsior*–*O. fragrans*, 11.9–44.4 Mya) were applied to generate the time tree through PAML (version 4.9.63) software [57]. The expansion and contraction of gene families were identified using CAFE5 (version 5.1.0) [58]. For estimation of polyploidization (WGD) events in olive we used the WGDI toolkit [59]. The OmicShare website (https://www.omicshare.com/tools) was used to perform GO and KEGG enrichment analyses.

### Identification of fatty acid and flavonoid biosynthesis pathway genes

To identify the members of fatty acid and flavonoid biosynthesis pathway gene families, the protein sequences of *A. thaliana* were downloaded and used as queries with Diamond software [60]. The requirements were as follows: (i) filtered for hits with an E value ≤1E−5, an alignment identity ≥50%, and an alignment coverage ≥50% of the query gene; and (ii) the presence of the same Pfam domains was used to query the *O. europaea* cv. ‘Leccino’ protein dataset with HMMER software by using an E-value <0.01. Multiple sequence alignment was carried out using MAFFT (version 7.471) [53] and phylogenetic analyses were performed using the Randomized Axelerated Maximum Likelihood (RAxML) program [61].

### Identification of structural variations between olive varieties

To investigative the structural variations in the genome between olive varieties, we used MUMmer4 (version 4.0.0) [62] to construct genomic comparisons (nucmer -t 30 —prefix = nucmer). Variants longer than 100 kb were filtered out. SV-influenced genes were defined by the distance (<2 kb) between the SVs. To classify the functions of SV-influenced genes, we performed GO analysis using the OmicShare website (https://www.omicshare.com/tools).

### Time-course transcriptomic and metabolomic detection for olive fruits

Five olive trees (*O. europaea* cv*.* ‘Leccino’) were cultivated in a common garden, the National Germplasm Repository of Olive of China (Longnan, Gansu, China), where all management practices were professional and optimal. The fruits were collected at 30, 60, 75, 90, 110, and 130 DAP. Each individual fruit was divided into two equal portions for transcriptome sequencing and metabolite detection. All samples were frozen with liquid nitrogen and stored at −80°C.

Total RNA was extracted and a library was constructed and sequenced on the Illumina NovaSeq platform by OE Biotech Co., Ltd (Shanghai, China). Clean reads were obtained using fastp software [63] and were aligned to the reference genome (T2T assembly in this study) using HISAT2 (version 2.0.4) [64]. Gene abundance was calculated using StringTie (version 1.3.4) [65] and normalized as FPKM (fragments per kilobase of transcript sequence per million base pairs sequenced). Candidate differentially expressed genes were generated using DESeq2 (version 3.11) [66]. The contents of various metabolites were determined and analyzed by Allwegene Tech Company (Beijing, China) using an UHPLC–MS/MS method [67–69]. The transcription factors within the modules were grouped into different families using iTAK software [70] and PlantTFDB (version 4.0) [71]. The finally co-expressed networks were visualized using Cytoscape (version 3.9.1) [72].

### Dual luciferase assay

The coding sequences of *MYC2* were amplified by PCR using specific primers; the product was 2019 bp in length (Supplementary Data Table S15) and introduced into the pCXSN vector by T4 DNA Ligase from New England Biolabs (Beijing, China). In addition, the promoter sequences of *BCCP2* and *FLS* were respectively cloned and integrated into the pGreen II 0800-LUC vector. Positive clones were sequenced by Tsingke Biotech Co., Ltd (Xian, China). The plasmids (200 ng) extracted from DH5α (*Escherichia coli*) of *BCCP2*-Luc, *FLS*-Luc, empty pCXSN vector and MYC2-PCXSN were respectively transformed into *Agrobacterium*-sensitive cells (GV3101). The reporter and effector (1:4) were co-transferred into the leaves of 1-month-old tobacco (*N. benthamiana*). The infected plants were cultured in darkness for 24 h, and then grown under light for 48 h. Firefly and *Renilla* luciferases were determined using a Dual Luciferase Reporter Gene Assay Kit from Yeasen Biotechnology Co., Ltd (Shanghai, China).

### Yeast one-hybrid assays

The G-box motif (5′-CACGTG-3′) with its flanking sequences (−232 and −268 bp) in the promoter of the *BCCP2* and the *cis*-sequence (5′-CACATG-3′) in the promoter (−1447 and −1483 bp) of *FLS* were duplicated three times by Tsingke Biotech Co., Ltd (Xian, China) and ligated to the pAbAi vector. The coding DNA sequence of MYC2 was introduced into the pGADT7 vector. Using the PEG/LiAc method [73], AD-MYC2 with proBCCP2-pAbAi or proFLS-pAbAi vectors were transformed into the yeast strain Y1H-Gold. Finally, the interaction was screened on SD-L/U solid medium supplemented with varying concentrations of AbA (aureobasidin A).

### Methyl jasmonate treatments

We used three concentrations (0.1, 1, and 5 mM) of MeJA to treat the olive fruits during the early stages of fruit development. Solution (0.1% ethanol and 0.1% Tween-20) without MeJA served as control group. Each group was set up with at least 50 fruits. After 1 month, the MeJA-treated and control fruits were collected and frozen immediately in liquid nitrogen for determination of the contents of fatty acids and flavonoids using LC–MS method by Allwegene Tech Company (Beijing, China). Total RNA was extracted using the CTAB protocol for subsequent RT–qPCR to quantify the expression of *MYC2*, *FLS*, and *BCCP2*.

## Supplementary Material

Web_Material_uhae168
