# Supplementary figures and images for: The gapless genome assembly and multi-omics analyses unveil a pivotal regulatory mechanism of oil biosynthesis in the olive tree
Source: Hortic Res. 2024 Jun 21;11(8):uhae168. doi: 10.1093/hr/uhae168 (PMC11300844; doi:10.1093/hr/uhae168)

*O. europaea* cv. Leccino

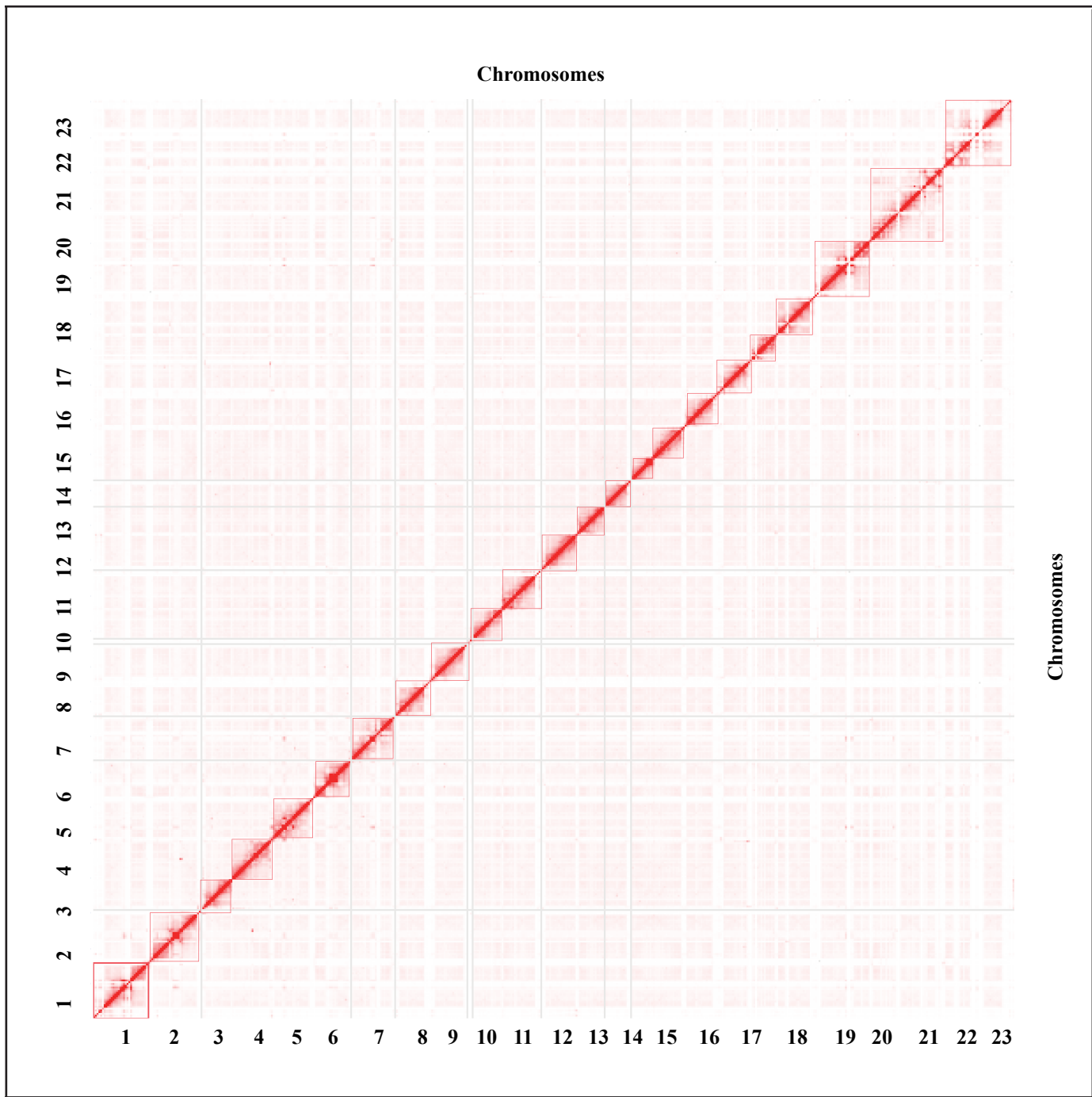

Supplement: Web_Material_uhae168 [file web_material_uhae168.zip › Supplemental Figure 1.pdf]

# Module-trait relationships

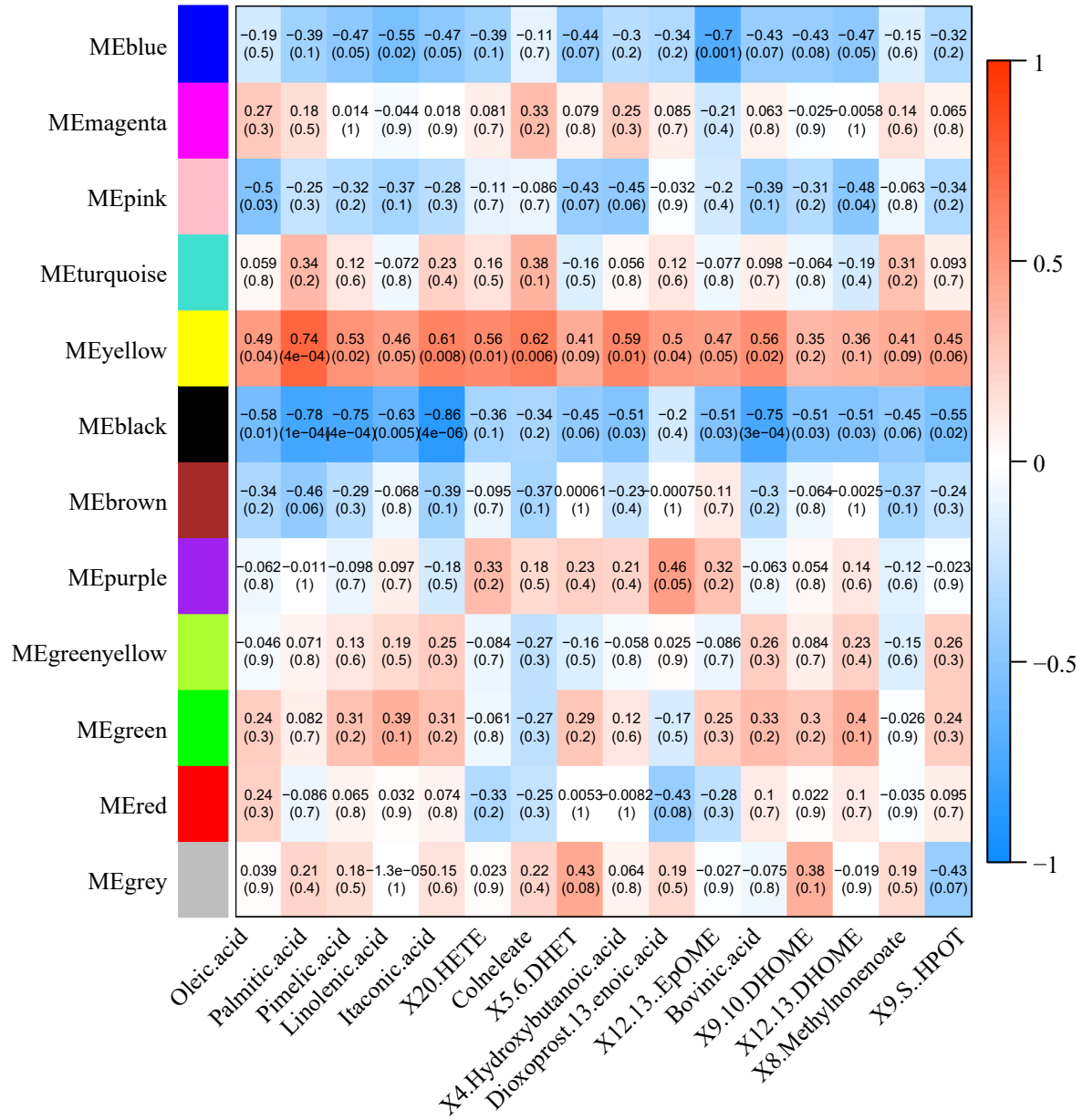

Supplement: Web_Material_uhae168 [file web_material_uhae168.zip › Supplemental Figure 10.pdf]

**Control**

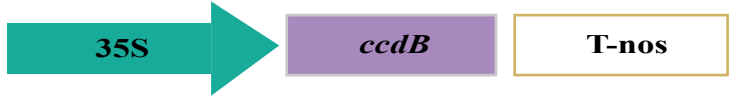

**Effector**

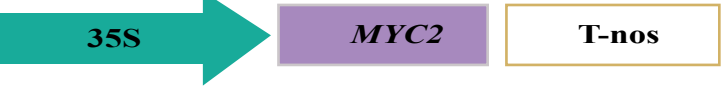

**Reporter**

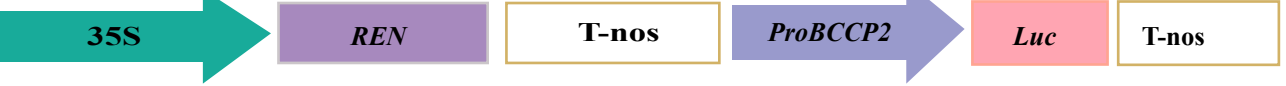

**Reporter**

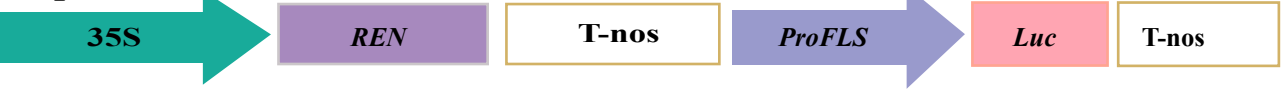

Supplement: Web_Material_uhae168 [file web_material_uhae168.zip › Supplemental Figure 11.pdf]

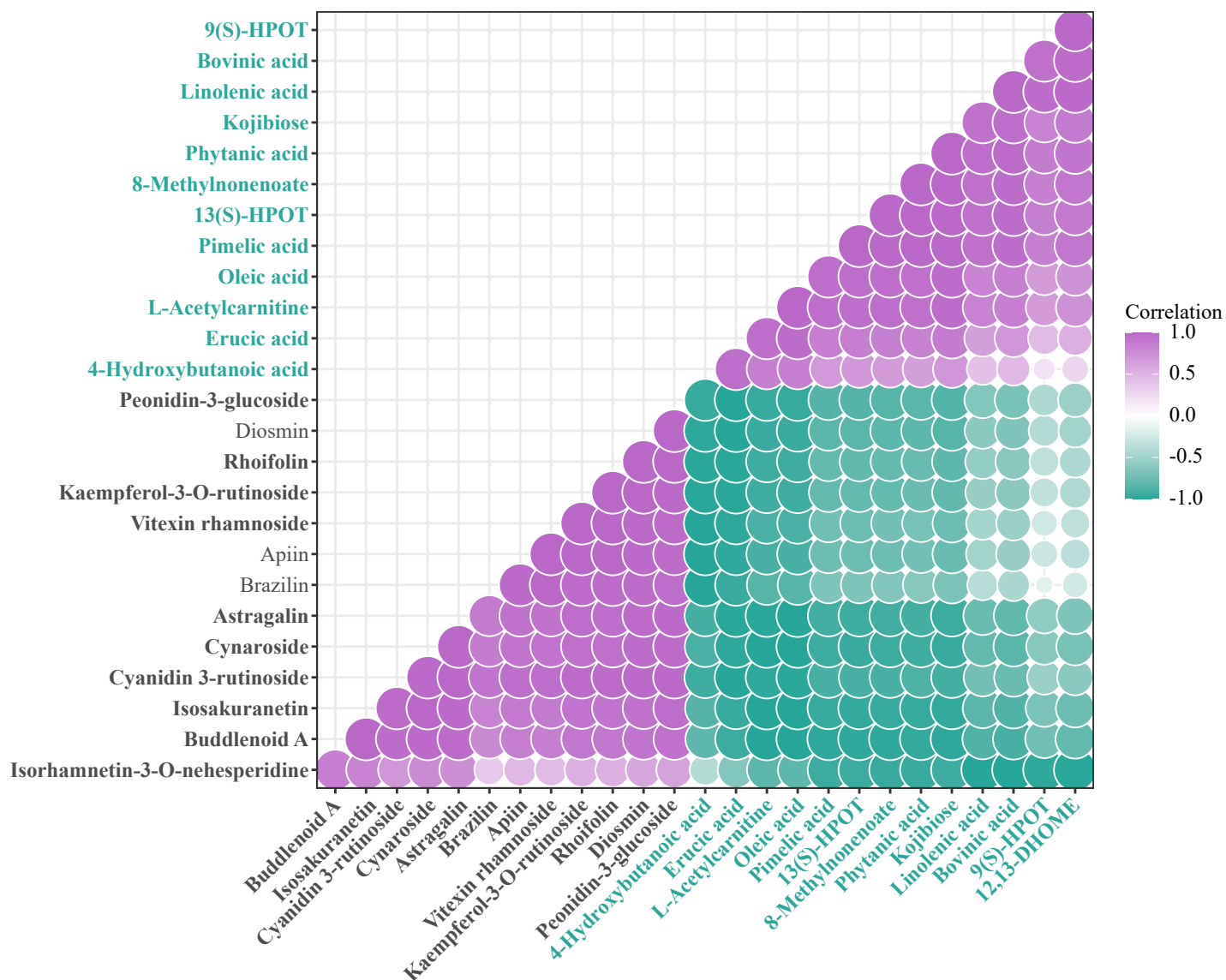

Supplement: Web_Material_uhae168 [file web_material_uhae168.zip › Supplemental Figure 12.pdf]

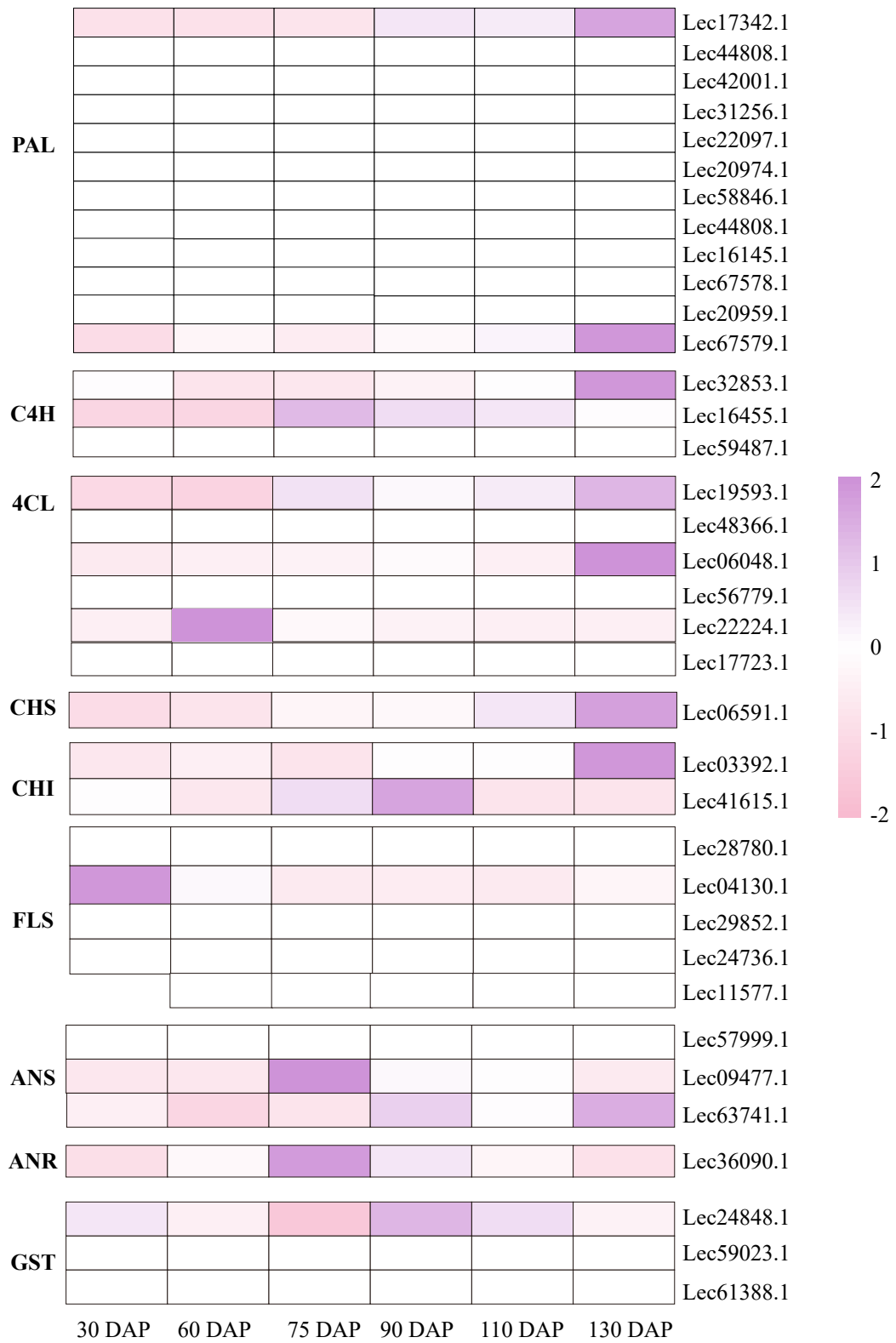

Supplement: Web_Material_uhae168 [file web_material_uhae168.zip › Supplemental Figure 13.pdf]

*O. europaea* cv. Coratina

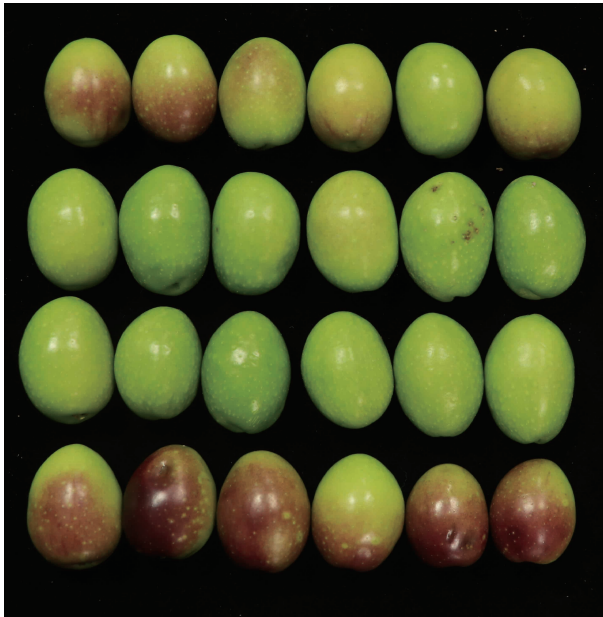

*O. europaea* cv. Ezhi8

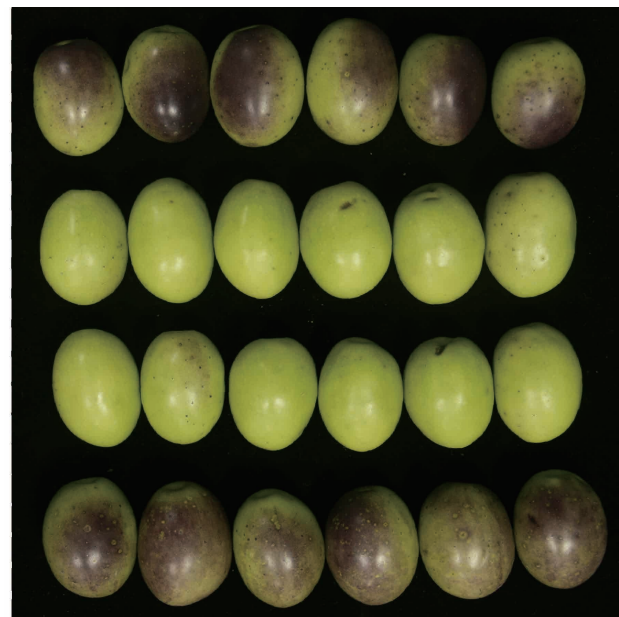

Control

0.1 mM MeJA

1 mM MeJA

5 mM MeJA

Supplement: Web_Material_uhae168 [file web_material_uhae168.zip › Supplemental Figure 14.pdf]

Pathway

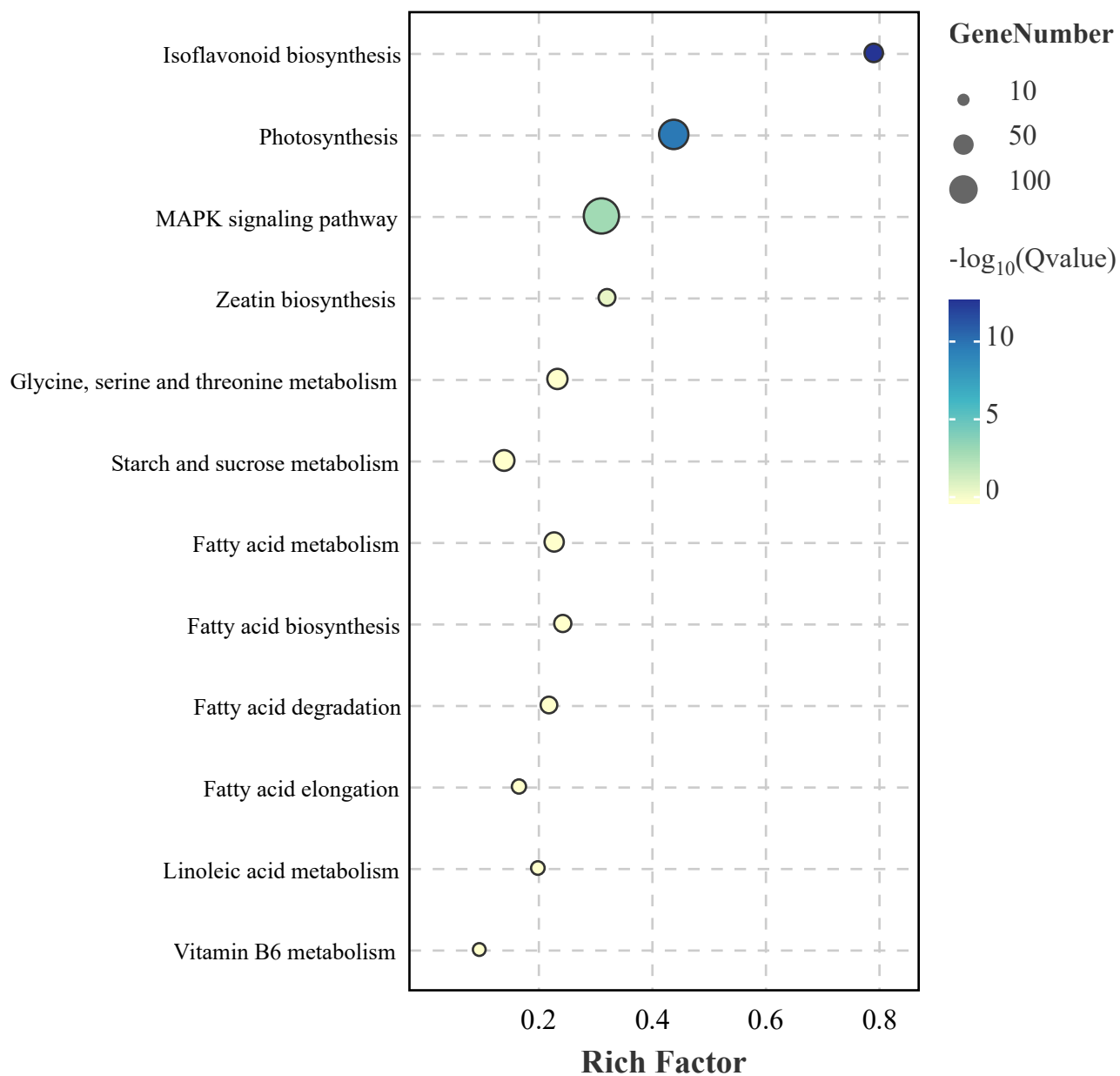

Supplement: Web_Material_uhae168 [file web_material_uhae168.zip › Supplemental Figure 2.pdf]

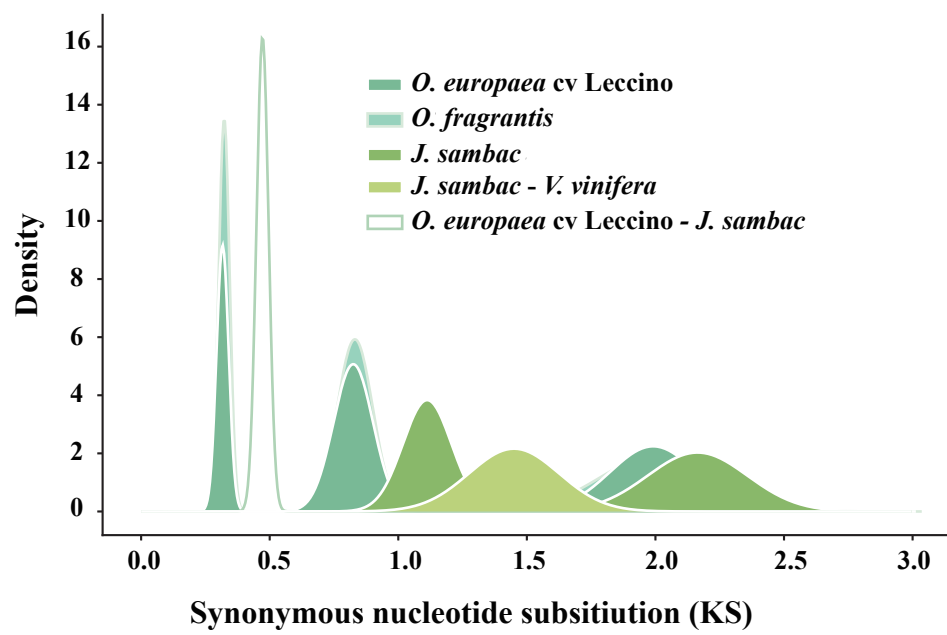

Supplement: Web_Material_uhae168 [file web_material_uhae168.zip › Supplemental Figure 3.pdf]

*O. europaea* cv. Leccino

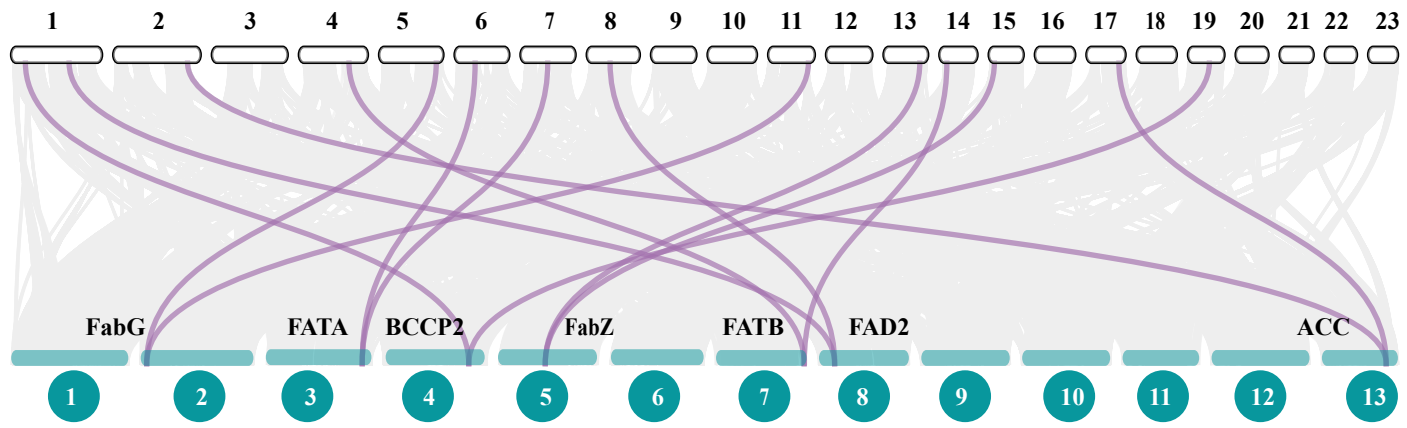

*J. sambac*

Supplement: Web_Material_uhae168 [file web_material_uhae168.zip › Supplemental Figure 4.pdf]

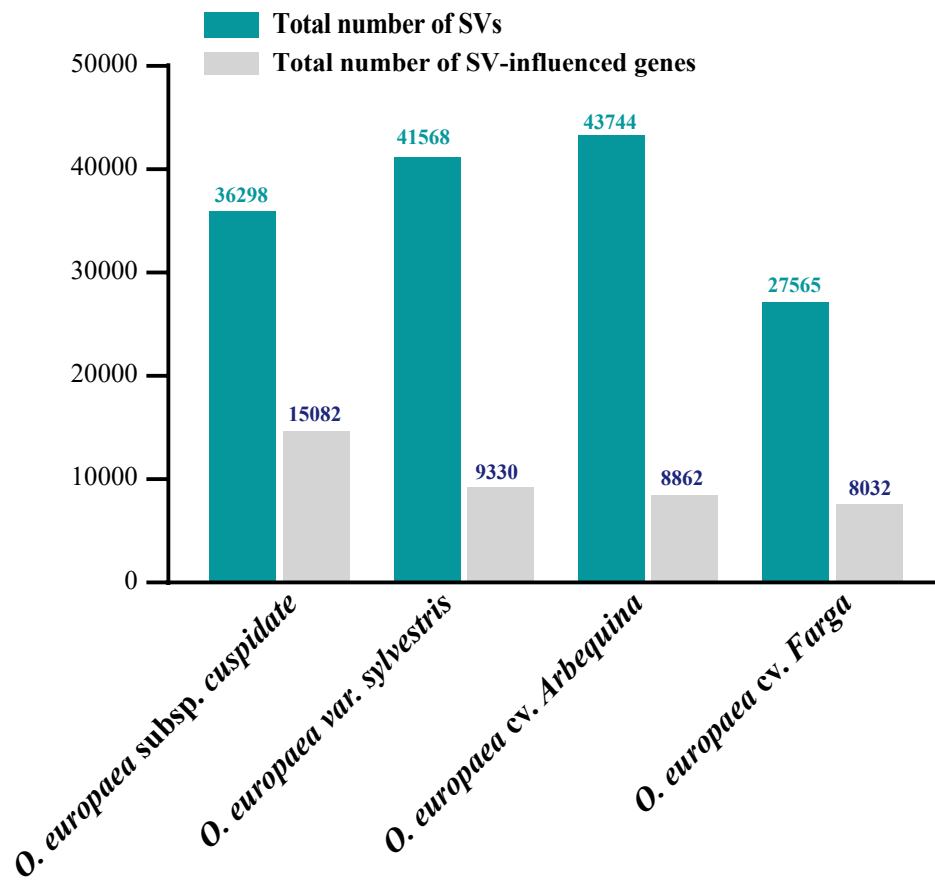

Supplement: Web_Material_uhae168 [file web_material_uhae168.zip › Supplemental Figure 5.pdf]

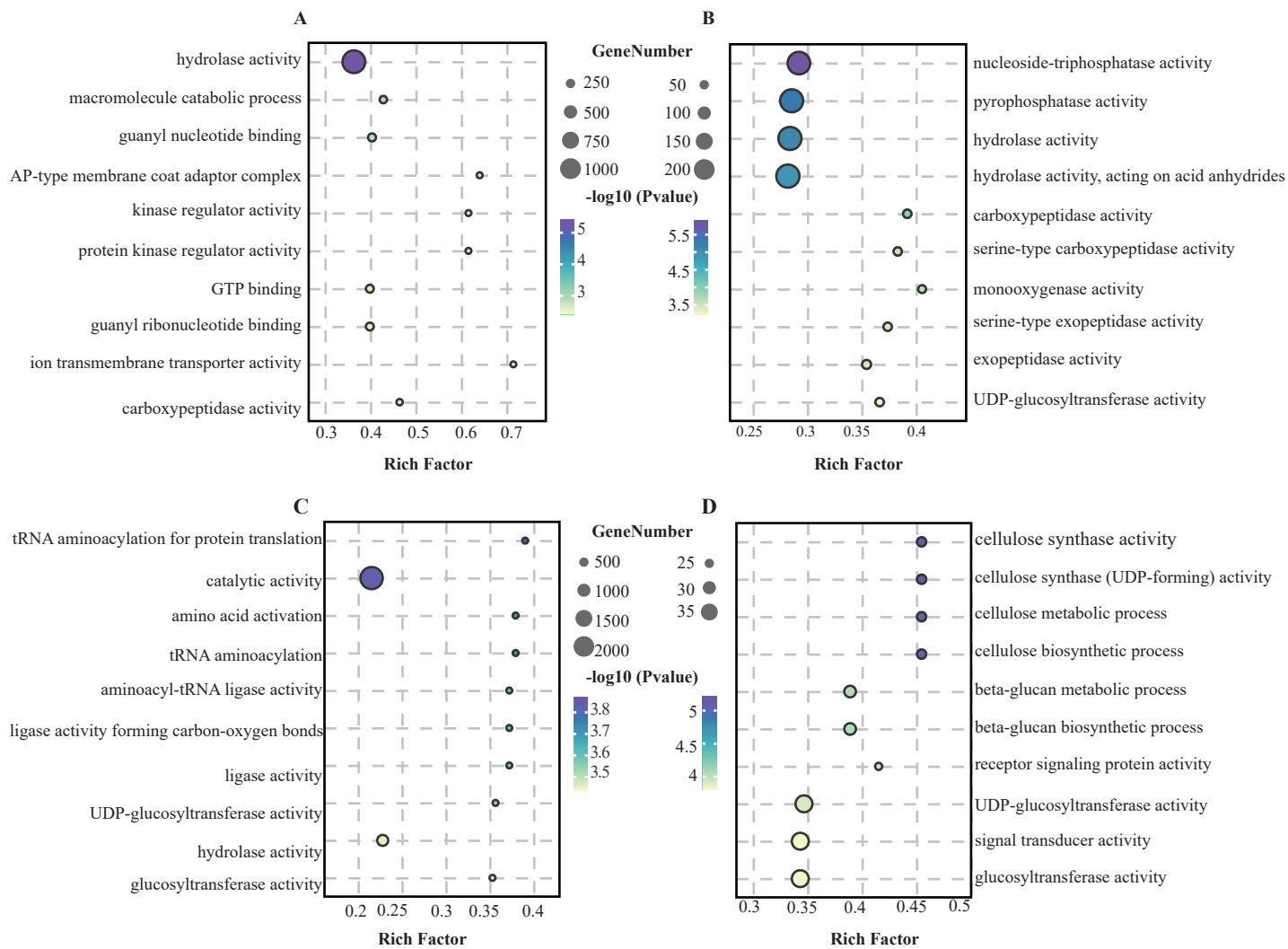

Supplement: Web_Material_uhae168 [file web_material_uhae168.zip › Supplemental Figure 6-.pdf]

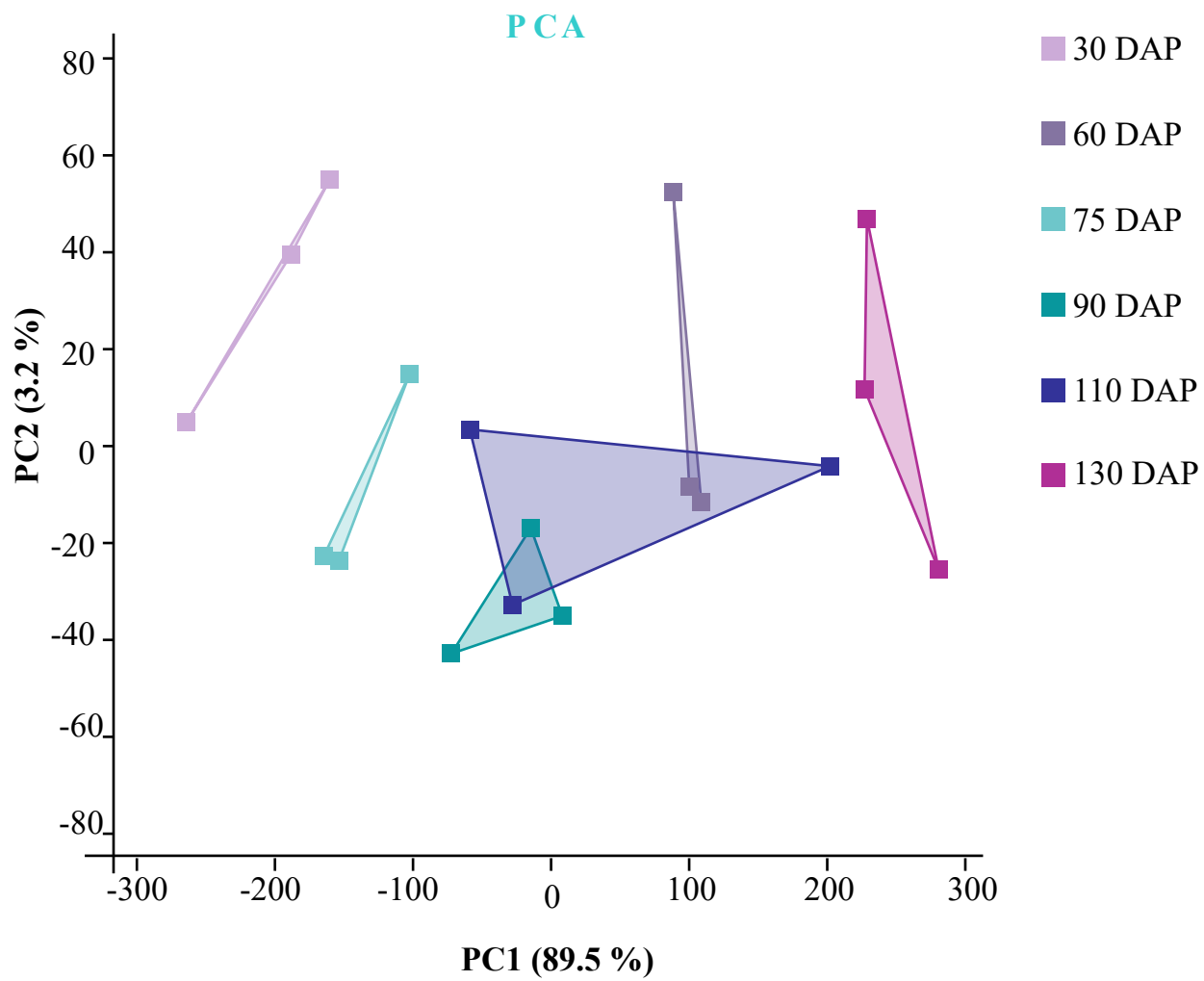

Supplement: Web_Material_uhae168 [file web_material_uhae168.zip › Supplemental Figure 7.pdf]

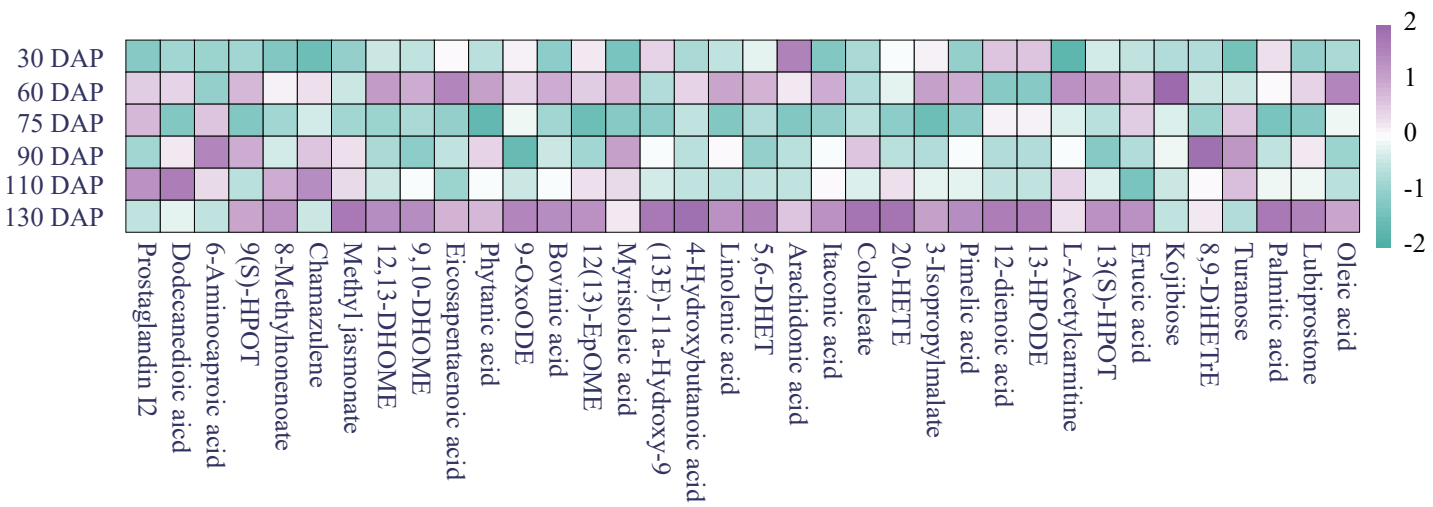

Supplement: Web_Material_uhae168 [file web_material_uhae168.zip › Supplemental Figure 8.pdf]

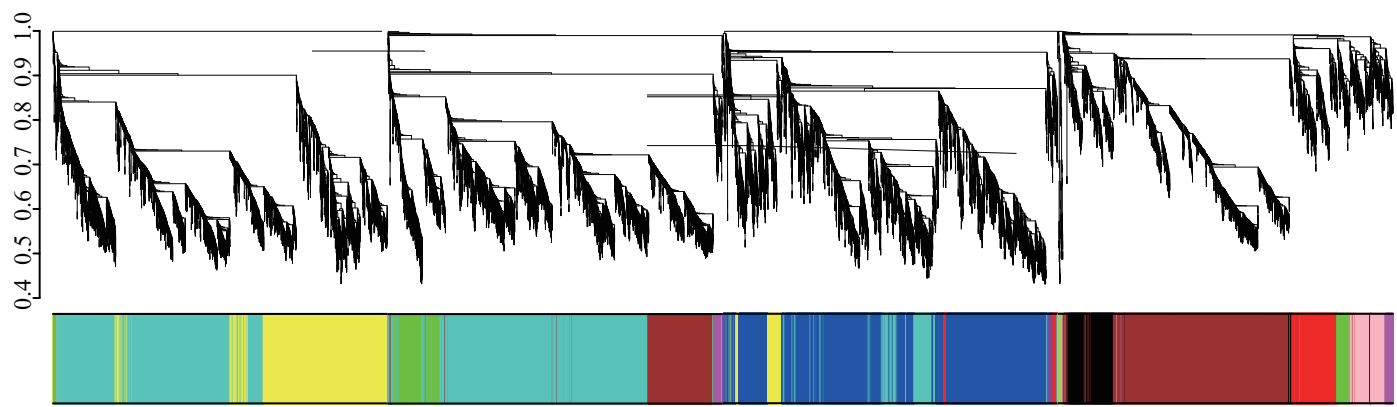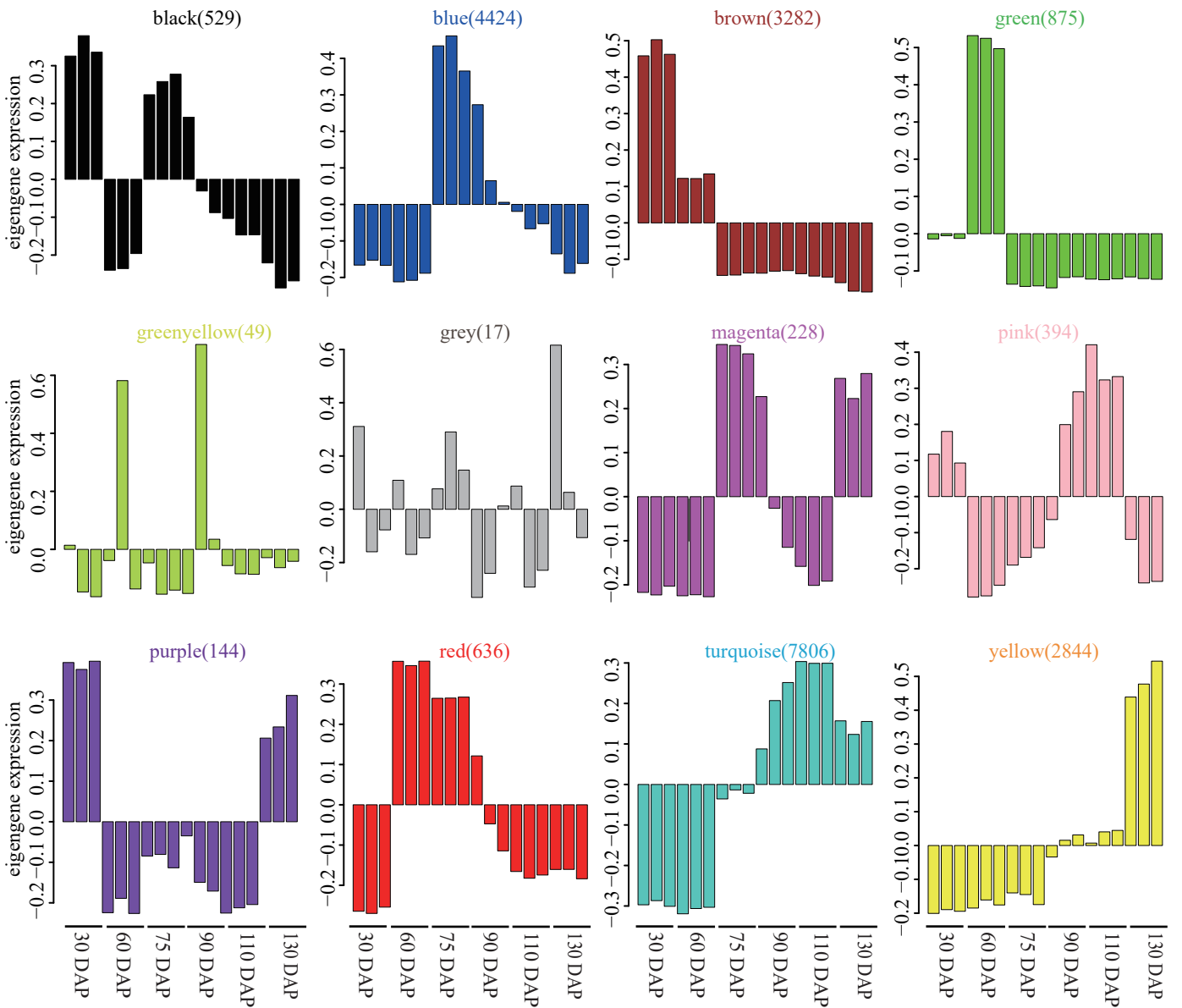

Supplement: Web_Material_uhae168 [file web_material_uhae168.zip › Supplemental Figure 9.pdf]
